# Pleuroperitoneal Leak: An Unusual Cause of Acute Shortness of Breath in a Peritoneal Dialysis Patient

**DOI:** 10.1155/2014/614846

**Published:** 2014-08-04

**Authors:** D. P. Ramaema, P. Mpikashe

**Affiliations:** Division of Radiation Medicine, Nelson R Mandela School of Medicine, University of Kwazulu-Natal, Private Bag Box 7, Congella, Durban, South Africa

## Abstract

*Introduction*. Pleuroperitoneal leak is an uncommon complication of continuous ambulatory peritoneal dialysis (CAPD), with an estimated incidence of 1.6%. It should be suspected in these patients when they present with recurrent unilateral pleural effusions and/or acute shortness of breath following dialysate infusion. *Case Presentation*. We present the case of a 25-year-old female patient who had acute hydrothorax as a result of pleuroperitoneal leak complicating continuous ambulatory peritoneal dialysis (CAPD), which was confirmed on peritoneal scintigraphy. *Conclusion*. Continuous ambulatory peritoneal dialysis patients presenting with acute shortness of breath and/or recurrent unilateral pleural effusion should be investigated with peritoneal scintigraphy to exclude pleuroperitoneal leak.

## 1. Introduction

Pleuroperitoneal leak is an uncommon complication of continuous ambulatory peritoneal dialysis (CAPD), with an estimated incidence of 1.6% [1]. Suspicion should arise when these patients present with recurrent unilateral pleural effusions and/or acute shortness of breath following dialysate infusion. Treatment is always individualised for each patient and can be conservative or surgical. In all cases, management commences with the interruption of peritoneal dialysis.

## 2. Case Presentation

**Figure 1:**
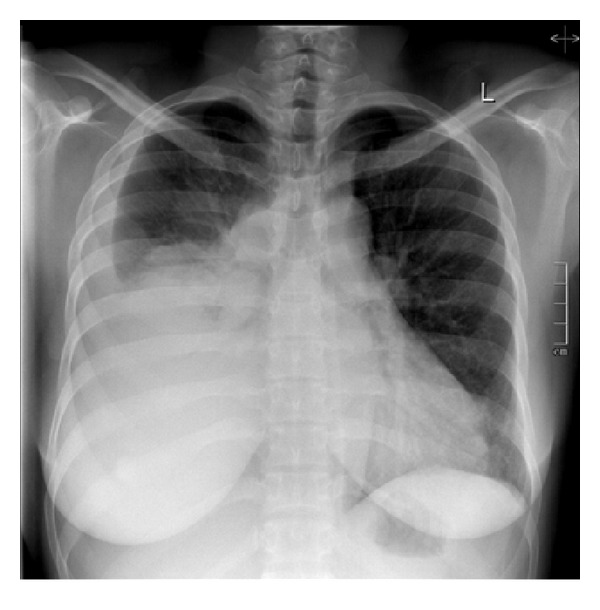
25-year-old female patient with right pleuroperitoneal leak following CAPD. Anteroposterior radiograph of the chest demonstrates large right pleural effusion with some underlying consolidation.

A 25-year-old female patient on longstanding continuous ambulatory peritoneal dialysis (CAPD), due to end stage renal disease (ESRD) complicating lupus nephritis, presented with respiratory distress following dialysate infusions. Repeated chest X-rays showed recurrent large right pleural effusions (Figure 1), requiring repeated therapeutic taps.

**Figure 2:**
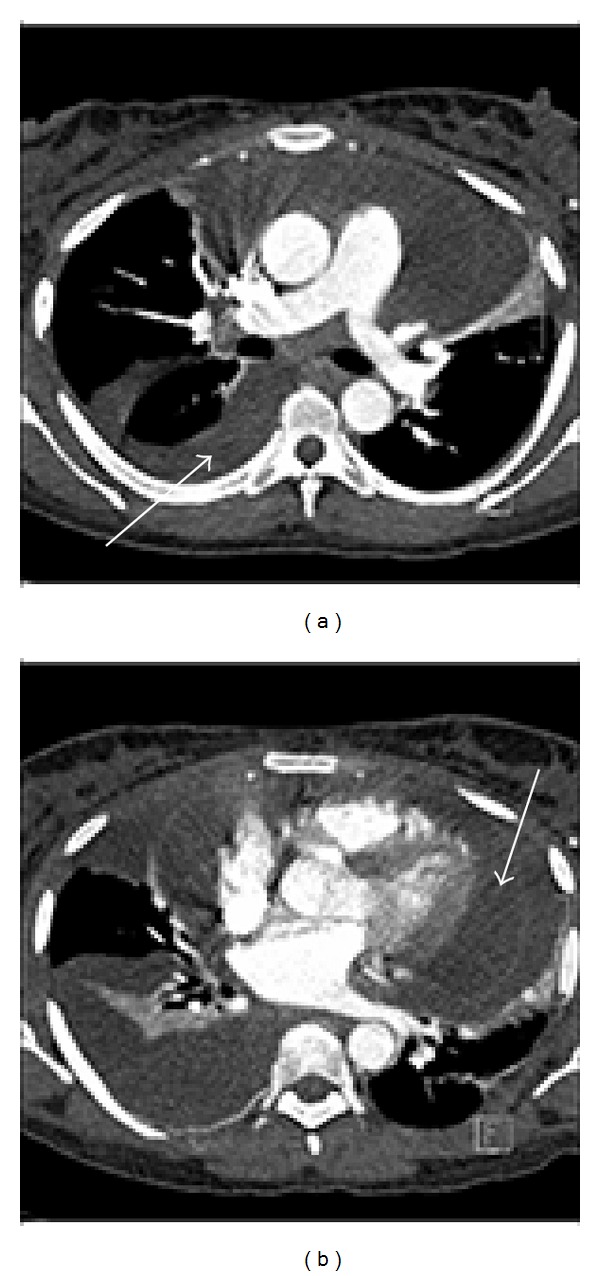
((a), (b)) 25-year-old female patient with right pleuroperitoneal leak following CAPD. Axial postcontrast CTPA image demonstrates right pleural effusion (arrow in (a)) and pericardial effusion (arrow in (b)).

As a result of her shortness of breath, a clinical diagnosis of pulmonary embolus (PE) was considered. Computed tomography pulmonary angiogram (CTPA) was performed and excluded pulmonary embolus. The CTPA confirmed large right pleural effusion with some underlying consolidation (Figure 2(a)) and small left pleural and large pericardial effusion (Figure 2(b)). Although the right pleural effusion was large and out of keeping with a systemic cause, the presence of the pericardial effusion and very small left pleural effusion suggested the diagnosis of fluid overload.

**Figure 3:**
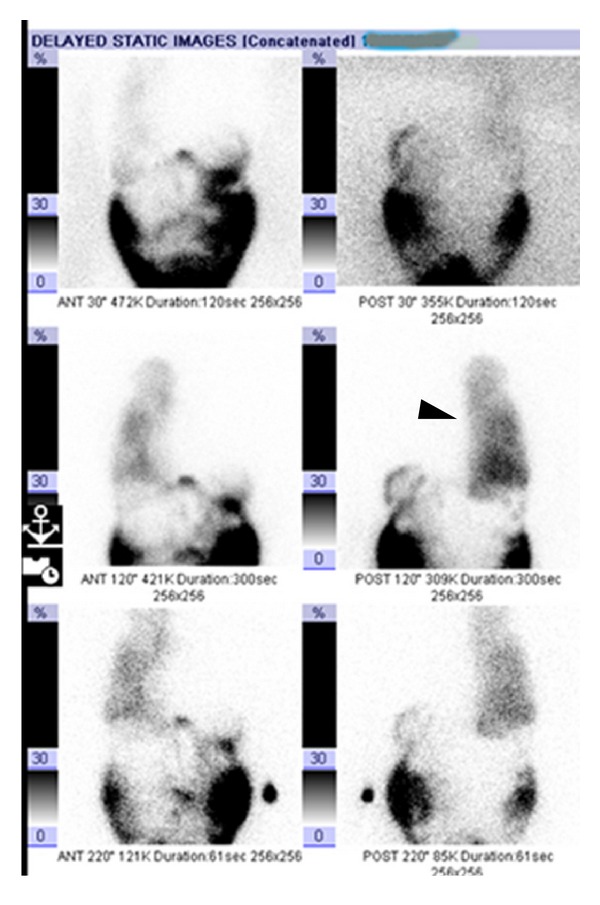
25-year-old female patient with right pleuroperitoneal leak following CAPD. Tc-99m MAA scan demonstrated progressive increase in activity in the right lung (arrowhead), confirming the diagnosis of pleuroperitoneal leak. The last image was taken at 3.5-hour delay.

However, the patient did not improve with clinical management for fluid overload and at that stage, the possibility of communication between the peritoneal and pleural cavity was considered. Peritoneal scintigraphy was performed using 3mCi Technetium Tc-99m Macroaggregated Albumin (Tc-99m MAA) injected into the abdominal dialysate port. The scan demonstrated a progressive increase in activity in the right lung, confirming the diagnosis of pleuroperitoneal leak (Figure 3). Peak activity values were measured to quantify the leak, which would be beneficial in treatment decision making as well as in monitoring her progress. Our patient was managed conservatively by changing to biweekly haemodialysis and monitoring her progress closely.

## 3. Discussion 

Pleuroperitoneal leak is a rare complication of CAPD and in automated peritoneal dialysis (APD) patients. Reported incidences range from 1.6% [1] to 2% [2]. The resultant pleural effusion is a transudative dialysate containing fluid with a low ADH, low cell count, and elevated glucose [3]. The mechanism for the leak is thought to be due to increased intra-abdominal pressure in the presence of underlying congenital or acquired diaphragmatic defect [4].

In most cases, as in our patient, the effusion is right sided. Autopsy and operative reports have suggested acquired anatomic diaphragmatic defects due to fluid filled blebs overlying tendinous diaphragm as a result of collagen fibre loss [5]. It is thought that the left side is partially protected by the heart and the pericardium, resulting in lower incidence of leaks. In view of lupus nephritis being the initial diagnosis in our patient, lupus serositis can be considered as a differential diagnosis; however this was not investigated. The diagnosis is generally suspected when a peritoneal dialysis (PD) patient develops recurrent unilateral pleural effusions and/or acute respiratory distress following dialysate infusion. The effusion has a relatively high glucose content compared to blood glucose, which is synonymous with peritoneal leakage [6]. Another clue to diagnosis is an unexplained low drainage volume of dialysate [2]. Definitive diagnosis can be obtained by peritoneal scintigraphy, as was the case in our patient. Diagnosis can also be made by CT scan with intraperitoneal (IP) radiocontrast admixed with the dialysis fluid. Several radiopharmaceuticals can be used for diagnosis, including Tc-99m MAA, as in our study, while, in another case, Tc-99m diethylene triamine pentaacetic acid (DTPA) was used [7] and colloid can also be used. Patients on PD with acute respiratory distress can be suspected to have PE, but this was ruled out with CTPA in our patient.

Treating pleuroperitoneal leaks varies and depends on the clinical condition of the patient, but in all cases, immediate interruption of the PD is required, which can be temporary or long term. Conservative treatment options include changing to haemodialysis, as in our patient, or using small volumes of modified PD techniques, with approximately 25% of cases responding to conservative treatment and eventually being reinstituted with CAPD [2]. Conservative interventional treatments, such as chemical pleurodesis, can be used [2]. Surgical interventions include open thoracotomy or newer less invasive thoracoscopic techniques, such as video-assisted thoracoscopic surgery (VATS), with or without diaphragmatic repair [2].

## 4. Conclusion

Continuous ambulatory peritoneal dialysis patients presenting with acute shortness of breath and/or recurrent unilateral pleural effusion should be investigated with peritoneal scintigraphy to exclude pleuroperitoneal leak.
